# liqDB: a small-RNAseq knowledge discovery database for liquid biopsy studies

**DOI:** 10.1093/nar/gky981

**Published:** 2018-10-24

**Authors:** Ernesto Aparicio-Puerta, David Jáspez, Ricardo Lebrón, Danijela Koppers-Lalic, Juan A Marchal, Michael Hackenberg

**Affiliations:** 1 Dpto. de Genética, Facultad de Ciencias, Universidad de Granada, Campus de Fuentenueva s/n, 18071 Granada, Spain; 2 Lab. de Bioinformática, Centro de Investigación Biomédica, PTS, Avda. del Conocimiento; 3 Department of Human Anatomy and Embryology, Institute of Biopathology and Regenerative Medicine, Excellence Research Unit “Modeling Nature” (MNat), University of Granada, Granada, Spain; 4 Biohealth Research Institute in Granada (ibs.GRANADA), University Hospitals of Granada-University of Granada, Spain; Conocimiento s/n, 18100 Granada, Spain; 5 Department of Neurosurgery, VUmc Amsterdam, Amsterdam, Netherlands

## Abstract

MiRNAs are important regulators of gene expression and are frequently deregulated under pathologic conditions. They are highly stable in bodily fluids which makes them feasible candidates to become minimally invasive biomarkers. In fact, several studies already proposed circulating miRNA-based biomarkers for different types of neoplastic, cardiovascular and degenerative diseases. However, many of these studies rely on small RNA sequencing experiments that are based on different RNA extraction and processing protocols, rendering results incomparable. We generated liqDB, a database for liquid biopsy small RNA sequencing profiles that provides users with meaningful information to guide their small RNA liquid biopsy research and to overcome technical and conceptual problems. By means of a user-friendly web interface, miRNA expression profiles from 1607 manually annotated samples can be queried and explored at different levels. Result pages include downloadable expression matrices, differential expression analysis, most stably expressed miRNAs, cluster analysis and relevant visualizations by means of boxplots and heatmaps. We anticipate that liqDB will be a useful tool in liquid biopsy research as it provides a consistently annotated large compilation of experiments together with tools for reproducible analysis, comparison and hypothesis generation. LiqDB is available at http://bioinfo5.ugr.es/liqdb.

## INTRODUCTION

Despite the well-established usage of blood and urine in disease detection and diagnosis, the term liquid biopsy does not appear in PubMed until 2011 in a work where breast cancer patients response to trastuzumab was monitored using circulating epithelial tumour cells (CETC) (1). Since then, liquid biopsy has become a rapidly growing research field based on the extraction of non-solid biological material such as blood, saliva, urine or cerebrospinal fluid that can be sampled in a minimally invasive way. From this material can then be extracted, among others: protein-bound RNA molecules, vesicles such as exosomes, cell-free DNA (cfDNA), circulating tumour cells (CTC) and platelets that can be used for clinical purposes. More specifically, genotypes and methylation states of extracted DNA molecules or the abundance of RNA molecules can be screened to search for non-invasive biomarkers that allow for early diagnosis, treatment monitoring, tumour staging, relapse risk assessment and prognosis (2).

Since microRNAs were discovered in humans in 2000, their functional role as post-transcriptional repressors has been extensively studied. MicroRNA expression levels are generally altered in several pathologies including cancer (3), so they hold great potential as disease biomarkers at tissue level. Furthermore, microRNAs have been detected in virtually all bodily fluids either within exosomes (4) or bound to proteins that protect them from RNAse activity (5), both of which increase their stability and therefore their detectability. If the release of most microRNAs is fairly random (6), the assumption is that intracellular changes can be detected in the different biofluids as well, which allows for potential applicability as diagnostic, prognostic and predictive biomarkers. In fact, several studies have already used this approach to propose miRNA-based biomarkers for different types of neoplastic (7,8), cardiovascular (9) and degenerative disease (10). Many of these studies rely on small RNA sequencing experiments but differences in sample collection, extraction, storage, processing, library preparation and sequencing method can have a strong impact on the abundance of detected miRNAs (11). Highly parametrized computational tools used for data analysis are yet another source of fluctuation in the obtained expression values. Note that most of these issues are not inherent to sequencing approaches, as other methods such as qRT-PCR are also affected by this panoply of possible confounding variables. Furthermore, no endogenous small RNA has been established to normalize abundance in plasma, although synthetic spike-in molecules have been proposed to address this problem (12).

In order to help to overcome the problems described above we developed liqDB, a database for small RNA expression profiles in bodily fluids. Unlike other literature-based resources such as miRandola (13) or ExoCarta (14), liqDB contains small RNA expression profiles of 1607 manually annotated samples from SRA, generated by means of a reproducible bioinformatics protocol. The database can be queried in different ways exploring 19 different biofluids or the impact of six variables like health state or RNA extraction method. Users can perform customised queries or compare uploaded data to sample sets from the database. Most important results are: downloadable expression matrixes, differentially expressed microRNAs and most stably expressed microRNAs. Visualisation of the output includes interactive RNA distribution boxplots and heatmaps (15). A strong increase in liquid biopsy small RNA research is to be expected over the next years and we are confident that liqDB can play a central role in organising, classifying and offering this information to researchers in the field.

## SCOPE AND WEB INTERFACE

### Scope

Currently, the database contains a total of 1607 coherently annotated sRNA-seq samples from 30 publically available SRA studies corresponding to 19 different biofluids. The sample annotations provided by the original authors were manually curated and unified to include relevant variables such as biofluid, gender, health state (healthy/not healthy), RNA extraction protocol, exosome isolation (yes or no) and RNA library preparation protocol, all of which can have an impact on the miRNA abundance acting as confounding variables for the variable of interest, usually related to the health state.

The database can be used and queried in five different ways:

- Browse studies: For each of the 30 SRA projects in the database, results were pre-calculated. Differential expression is calculated whenever possible for the relevant variables (mostly health state and gender although RNA extraction, library preparation methods and different biofluids were analysed for some studies). Additionally to the miRNA expression profiles, other results are generated (see *Result Page* below for more details). Frequently, users are interested in a particular project focused on a specific cancer or biofluid and this information can be quickly accessed in this way.
- Search samples: Users can customize a set of samples by selecting query values for 6 variables and a threshold number of miRNA-mapping reads in order to improve confidence in the results. The *Basic statistics* section can help users find the most adequate threshold for their desired query. At a second stage, the preselected set of samples can be manually refined. Most highly expressed, most fluctuating and most stably expressed microRNAs can be obtained, among other features. The downloadable expression matrix can be used for further downstream analysis (see *Result Page* below for more details).
- Search miRNAs: Users interested in one particular miRNA can search it and analyse its expression values as a function of the different variables.
- Compare two datasets: Similarly to the selection of one set, the user can define two different sets of samples. Additionally to the general output results, differentially expressed microRNAs between the two sets will be calculated.
- Compare with user data: This tool allows comparison of a selected set of samples from the database to user-provided samples. Users should first profile their sequencing reads input file using sRNAbench from the sRNAtoolbox server (16). Subsequently, the job IDs can be used as input as well as relevant query variables to generate a standard result page including differential expression between database and uploaded samples.

### Results pages

The standard results page includes the sections described below.

- Overview/query results: The full annotation of all selected samples.
- miRNA profiles: a sortable and searchable expression matrix with adjusted RPM values (Reads Per Million), a boxplot of the 20 most abundant microRNAs, a pie-chart showing the relative frequency of the 10 most abundant microRNAs, the 20 microRNAs with highest coefficient of variation (CV) and the 20 microRNAs with lowest CV. The CV is calculated as the standard deviation of the adjusted RPM expression values divided by the mean value. It is a standardized measure of the dispersion of RPM values which does not depend on the magnitude and therefore allows to compare the dispersion of highly and lowly abundant microRNAs. The expression of microRNAs with lowest CV is less affected by the different variables in the analysis and could be used as reference microRNAs to normalize or standardize qPCR validation experiments.
- sRNA types distribution: Proportions of reads assigned to each of the different small RNA types (miRNA, tRNA, yRNA, ribosomal RNA, etc) are shown in a table and the 10 most frequent categories are depicted in boxplots. The category ‘Un-assigned’ contains genome mapped reads which could not be assigned to any annotation.
- Species Distribution: A high number of reads that cannot be mapped to the genome can indicate contamination or the presence of genetic material from symbionts or parasites. In order to address this question, liqDB summarizes the mapping to the human genome, virus and bacteria collections showing the relative frequencies of reads assigned to the different species. If a read maps to more than 20 loci in the genome, it will not be used for expression profiling. Those reads get the label ‘HR’ for highly redundant. ***hsa_HR*** will therefore refer to the relative frequency of reads that map more than 20 times to the human genome. Furthermore, a read can map with the same quality (number of mismatches and length) to different indexes. In this cases, a new category is generated mentioning all genomes separated by ‘-’. For example ***human_virus-hsa***will refer to the number and percentage of reads that map both to the human genome and the virus collection.
- Download: Expression matrices are available for download as well as a zip file with the complete analysis.
- Differential Expression: if available, pre-calculated study specific comparisons or generated from user-provided groups are displayed. Differentially expressed microRNAs are calculated using two-sided t-test on Reads Per Million values. Subsequently, p-values are corrected for multiple testing applying the Bonferroni procedure. Boxplots of the most abundant differentially expressed microRNAs are displayed and a link to a heatmap is provided. Files for the complete analysis can be found in the download section. Note that the output for ‘Compare two datasets’ (user selected sets) will always contain one pairwise comparison, while the differential expression section in ‘Browse studies’ might contain several pairwise comparisons if more than two groups exist for one variable (e.g. SRP061240 with three different cancer types and healthy controls).

The microRNA search generates one output page with several boxplots depicting the expression of the microRNAs as a function of the different variables.

## DATA AND METHODS

### Database construction

All expression values and metadata were uploaded to a MySQL database. The interactive web interface was implemented using the Django framework together with Bootstrap, javascript and the plotly package (Plotly Technologies, 2015, Collaborative data science, https://plot.ly) for interactive data visualization. A backend java program connected to the database calculates and prepares the raw results files. Apache2 was chosen as HTTP webserver.

### Data collection and processing

Suitable data was searched using the NCBI based SRA (short read archive) repository and publications included in PubMed. The data was downloaded in sra format and converted with *fastq-dump* to standard fastq format. For expression profiling, sRNAbench, the successor program of miRanalyzer (17) was used. After removing adapter or barcode sequences, the obtained clean reads are collapsed into unique reads (UR) assigning a read count (RC) to each unique read sequence. By means of bowtie1 (18) the collapsed reads are mapped simultaneously to the human genome (GRCh38, patch 10), a collection of bacteria (Bacteria Ensembl, Release 39) as well as to human virus sequences (Human virus from EnsemblGenomes) and only the best mapping reads are retained as described before (17). One mismatch within the 19 nt seed region was allowed in the mapping process.

The genome mapped reads are then assigned successively to several reference libraries in a hierarchical way in the exact order described below. After each step, reads can only be assigned to one library in order to avoid cross-library matches. For example, after mapping miRNA reads, those are removed and cannot be assigned to tRNAs or other small RNA annotations.

- ***miRNAs***: miRBase v22 (19) and miRGeneDB v.2 (Fromm *et al.*, MirGeneDB2.0: the curated microRNA Gene Database. *bioRxiv*, https://doi.org/10.1101/258749). We produced a merged annotation using miRBase names adding miRGeneDB sequences if those are not annotated in miRBase. Human and several virus annotations are used for profiling.
- tRNAs: GtRNAdb, a genomic tRNA database (20)
- vault-RNA, yRNA and guide RNAs extracted from RefSeq (21)
- Non-coding RNAs from Ensembl (22)
- Non-coding RNAs from RNAcentral release 9 (23)
- RNA sequences of coding genes from Ensembl (Release 91) (22)

Please note that no further filtering of the miRBase reference sequences was performed which implies that, very likely, false positive miRNAs exist in the database. An example of this is miR-1246, which turned out to be a U2 small nuclear RNA (RNU2-1) fragment useful to discriminate tumours from controls (24).

### Expression values and relative frequencies

The microRNA expression values are calculated based on raw read counts adjusted for multiple mapping. The expression matrixes in RPM or adjusted raw read counts can be downloaded. The latter is the required format for most differential expression packages like DEseq (25) and edgeR (26).

## WORKING EXAMPLES

### The influence of library preparation protocols on the microRNA profiles

The influence of different library preparation protocols on plasma-derived exosomal RNA frequencies was first studied in 2013 by Huang *et al.* (27). These authors report that the five most abundant microRNAs account for 49% of all miRNA mapped reads. Since this study is available at liqDB, we can replicate these results by simply clicking on *Browse liqDB, Browse studies*, finding this study (SRP020486) and then navigating to the *miRNA Profiles* tab. In the miRNA profiles table it can be confirmed that liqDB lists the same five miRNAs as the most frequent to make up for 46% of miRNA mapped reads. Small differences are likely due to different miRBase versions as well as the inclusion of miRGeneDB in liqDB. Figure 2A shows the differentially expressed miRNAs detected for this study, i.e. the miRNAs which are highly influenced by the library preparation protocol. Since there are only two Illumina samples, statistical significance can only result from Bioo Scientific (NEXTflex) vs. NEBnext protocol comparisons. Hsa-miR-128 is the most expressed microRNA that is highly affected by the protocol. Figure 2A gives a median RPM for NEBnext protocol of 237 658 (243 837 in the original article) while NEXTflex only yields 4655 (4569) RPM, nearly 2 orders of magnitude below. RPM values from liqDB and the original article are therefore nearly identical.

**Figure 1.**
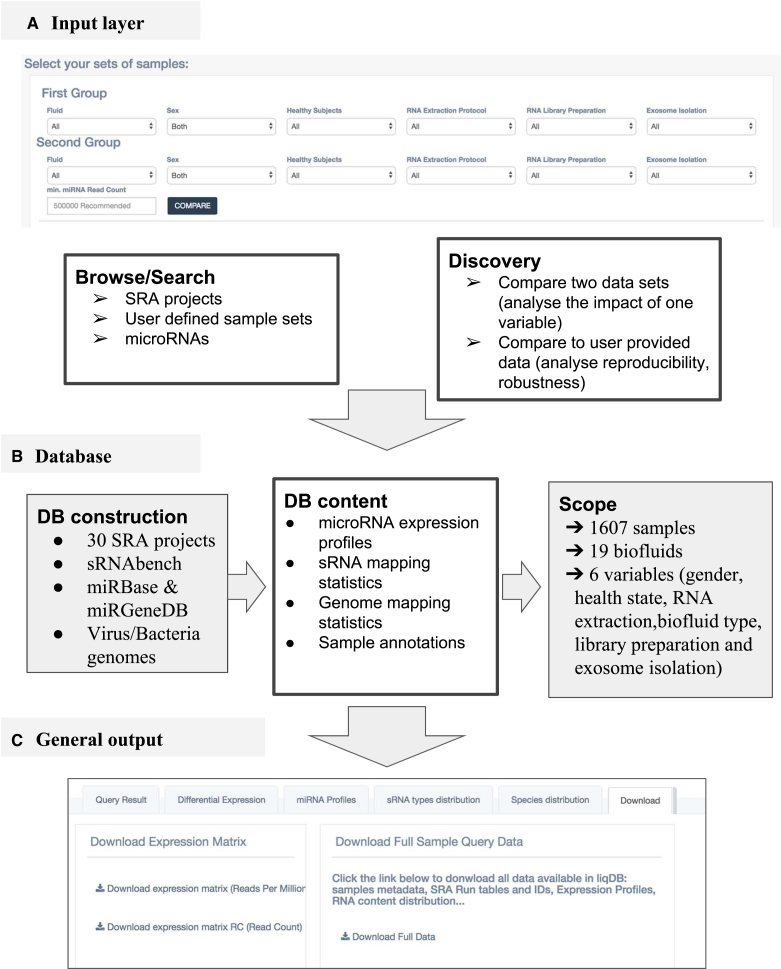
A schematic overview of liqDB. (**A**) the database can be queried in five different ways, either by browsing pre-calculated content or by instant processing of user-defined sets of samples. (**B**) liqDB was populated with 1607 samples from 19 different biofluids. The profiling of the data is carried out by means of sRNAbench (16) using both miRBase and miRGeneDB as annotations. (**C**) The general output consists of several sections, including miRNA profiles, differential expression (only if applicable) and download (shown in the figure).

**Figure 2.**
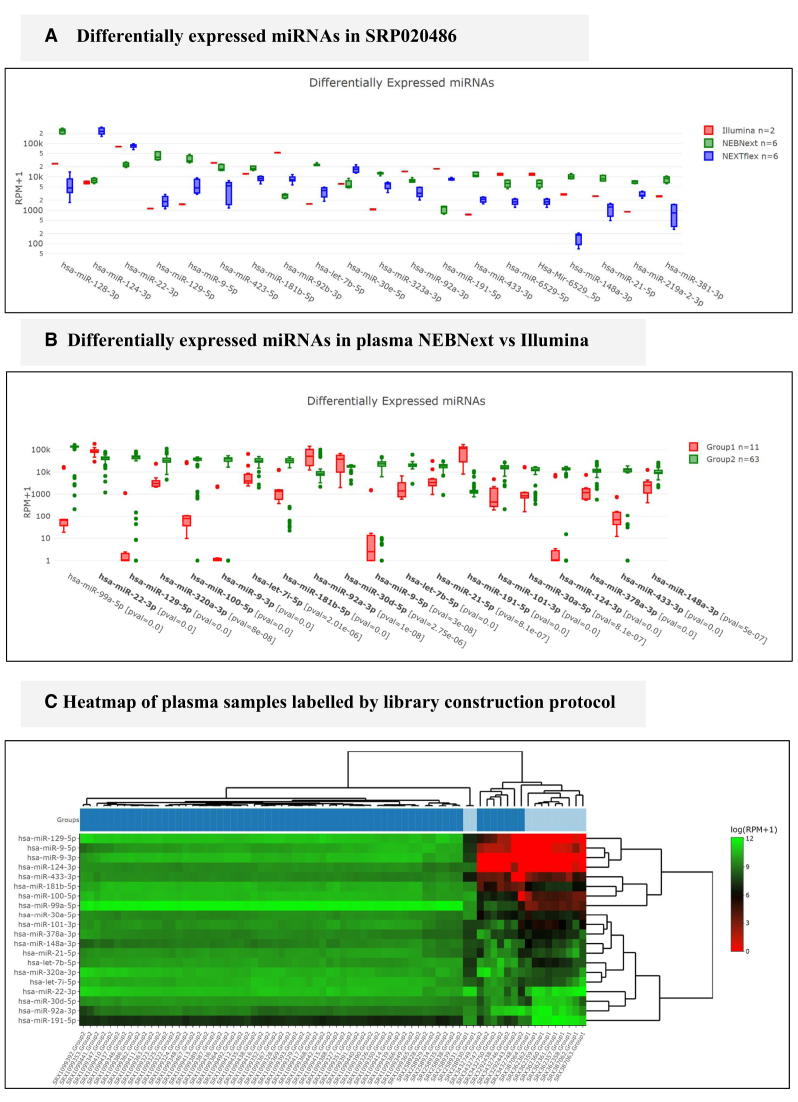
(**A**) Differentially expressed genes for three different library preparation protocols. (**B**) Differentially expressed miRNAs between NEBnext and Illumina protocol using all plasma samples in liqDB. (**C**) Most Illumina samples (bright blue) cluster together except for two of them in the middle of the NEBnext cluster (dark blue).

However, since many more samples are included in liqDB, we can extend this analysis to a larger query in order to increase confidence in the results. For instance, we can select plasma from all healthy subjects that used miRNeasy RNA extraction and compare the samples that applied Illumina library preparation protocol to those that used NEBnext. To do so, we go to *Compare datasets* > *Compare two sets of samples* and we set the selectors (see Figure 1A) for both groups following the desired parameters. We included a minimum threshold of 200 000 miRNA reads to increase the robustness of the outcome and then proceeded with all samples. While this comparison yields three out of six most abundant microRNAs in common with the SRP020486 results, it also reveals that 11 out of the 20 most abundant microRNAs are differentially expressed. This confirms again that the miRNA abundance in plasma is strongly affected by the library preparation protocol. The hierarchical clustering (see Figure 2C) shows as well that samples are grouped mostly by library protocol, although there is also one cluster with NEBnext and Illumina samples which indicates the existence of other variables that influence the miRNA expression profiles.

Recently, the influence of sample processing in miRNA sequencing was evaluated on a large scale study among nine different laboratories (28) confirming the strong impact of library preparation and other variables.

### Finding least variable miRNAs in serum samples

**Figure 3.**
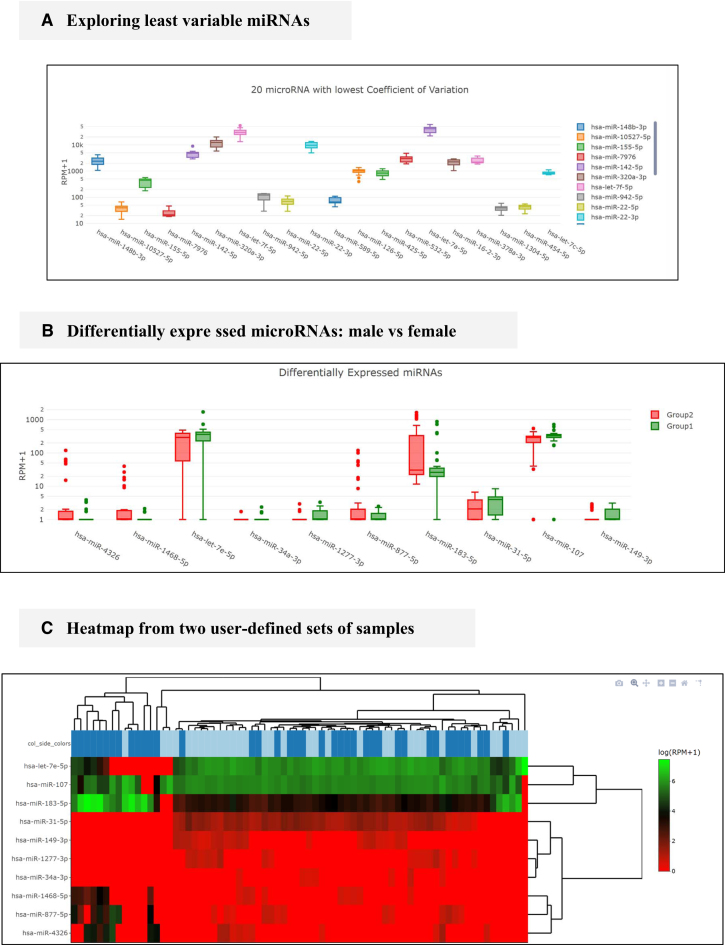
Examples from the web interface. (**A**) Boxplot of least variable miRNAs, candidates to control downstream validation. (**B**) Example of differentially expressed miRNAs boxplots. (**C**) Example of heatmap displaying clustering of plasma samples using differentially expressed miRNAs. Men are marked in dark blue and women in bright. Generated using heatmaply (15).

For some downstream validation experiments such as qRT-PCR, it may be relevant to know which miRNAs are most stably expressed in order to use them as reference. In this case, we queried the database for serum samples using the following workflow: *Browse liqDB* > *Search samples*; then a series of selectors are displayed. For these, we chose *serum* for Fluid, *Both* for Sex, *True* for Healthy Subjects, *miRNeasy* for RNA extraction Protocol and *NEBnext* for RNA Library preparation and then clicked Filter. To avoid effect of other confounding factors, we limited the analysis to only one extraction and one library protocol. Subsequently, we kept all samples for analysis by clicking on *Proceed with all samples*. In the results page, we then navigate to the *miRNA Profiles* tab where the last graph will show the miRNAs with the lowest variability for the specified query (Figure 3A).

### Comparing two sets of samples: plasma of men versus plasma of women

In order to compare two sets of samples in the most realistically possible way, potential confounding variables should be controlled for both sets. In this case we analysed differences in plasma between men and women. To do so, we first navigated to *Compare Datasets > Compare two sets of samples*. Similarly to the previous example, we will find two sets of selectors: one for the first group of our comparison and another for the second (See Figure 1A). For both selectors, we set Fluid to *plasma*, Healthy subjects to *True*, Extraction protocol to *miRNeasy* and Library protocol to *NEBnext*; for Group 1 Sex will be *male* and for Group 2 *female*. Once in the page of results, we navigated to the *Differential Expression* tab.

The boxplots graph (see Figure 3B) displays the 10 differentially expressed miRNAs with the highest expression values and then individual samples can be clustered and visualized in a heatmap (see Figure 3C).

## CONCLUSIONS AND OUTLOOK

Given that miRNAs are highly stable and relatively detectable in most biofluids, it is safe to assume that they will play important roles in the fast growing field of liquid biopsy. Many projects use high-throughput sequencing approaches in the exploratory phase of biomarker discovery making the data publicly available through repositories such as SRA or GEO. In order to structure, organize and unify this vast amount of information into an interactive database, major problems like incomplete or inconsistent sample annotations need to be solved. liqDB is the first database that provides researchers from the liquid biopsy field with browse- and downloadable coherently annotated datasets generated using the same bioinformatics protocol. Furthermore the database allows the comparison to external data therefore enabling the generation and testing of new hypothesis. We also encourage researchers to share their data through SRA and submit the accession to liqDB or just to point out any overlooked SRA projects that might be suitable for inclusion.

In the short term, there are three main improvements planned. First, other small RNA derived sequences like isomiRs or fragments of tRNA, yRNA or vault-RNA molecules will be included in liqDB as they all might have biomarker potential (29). Secondly, we will add different quality related flags to the sample annotations so the user can decide to exclude lower quality samples (30). And finally, since differential expression is one of the key analysis for gene expression data, we will improve this feature by adding online support for DEseq (25) and EdgeR (26). These additions will be useful for exploratory analysis of the data as well as for the analysis of confounding variables.

In summary, we anticipate that liqDB will be a useful tool for liquid biopsy researchers as it can help to develop standardized and stable protocols opening the door to reproducible reanalysis, realistic comparison and hypothesis generation, important tasks to avoid unnecessary validation of defective biomarker candidates which could prevent the discovery of actually useful biomarkers.
